# Human patient derived organoids: an emerging precision medicine model for gastrointestinal cancer research

**DOI:** 10.3389/fcell.2024.1384450

**Published:** 2024-04-04

**Authors:** Sicheng Yan, Yuxuan He, Yuehong Zhu, Wangfang Ye, Yan Chen, Cong Zhu, Fuyuan Zhan, Zhihong Ma

**Affiliations:** ^1^ Huzhou Key Laboratory of Molecular Medicine, Huzhou Central Hospital, The Affiliated Central Hospital of Huzhou University, Huzhou, China; ^2^ School of Basic Medicine College, Zhejiang Chinese Medical University, Hangzhou, China; ^3^ Department of Colorectal Surgery, Huzhou Central Hospital, The Fifth School of Clinical Medicine of Zhejiang Chinese Medical University, Huzhou, China

**Keywords:** gastrointestinal cancers, organoid, 3D model, CRISPR/cas9, personalized medicine

## Abstract

Gastrointestinal cancers account for approximately one-third of the total global cancer incidence and mortality with a poor prognosis. It is one of the leading causes of cancer-related deaths worldwide. Most of these diseases lack effective treatment, occurring as a result of inappropriate models to develop safe and potent therapies. As a novel preclinical model, tumor patient-derived organoids (PDOs), can be established from patients’ tumor tissue and cultured in the laboratory in 3D architectures. This 3D model can not only highly simulate and preserve key biological characteristics of the source tumor tissue *in vitro* but also reproduce the *in vivo* tumor microenvironment through co-culture. Our review provided an overview of the different *in vitro* models in current tumor research, the derivation of cells in PDO models, and the application of PDO model technology in gastrointestinal cancers, particularly the applications in combination with CRISPR/Cas9 gene editing technology, tumor microenvironment simulation, drug screening, drug development, and personalized medicine. It also elucidates the ethical *status quo* of organoid research and the current challenges encountered in clinical research, and offers a forward-looking assessment of the potential paths for clinical organoid research advancement.

## 1 Introduction

Gastrointestinal (GI) tumors are a common group of malignant tumors, including gastric, colorectal, esophageal, pancreatic, hepatocellular, and cholangiocellular cancers (Liu et al., 2022; Shi et al., 2022). Among them, gastric cancer (GC) and colorectal cancer (CRC) are the most prominent GI cancers (Weng et al., 2019; Huang et al., 2023). According to the global Cancer Statistics in 2020, there were about 19.3 million new cancer cases worldwide, including more than one million new cases of GC and 769,000 deaths. Globally, GC ranks fifth in incidence and fourth in mortality (Xia and Aadam, 2022). CRC is the third most common cause of cancer mortality worldwide, with more than 1.85 million cases and 850,000 deaths annually (Biller and Schrag, 2021). Furthermore, alterations in lifestyle behaviors, heightened levels of stress, and bacterial infections, *etc.*, have contributed to the escalating prevalence of GI cancers, resulting in a younger age of onset and a substantial impact on individuals’ overall health and wellbeing (Tong et al., 2021).

The pathogenesis of GI tumors is a complex process caused by the interaction of genetic, environmental, and other factors (Tong et al., 2021). Genetic variations in key genes s such as tumor suppressors and oncogenes play a significant role in the transformation of normal cells into cancerous ones (Al-Ishaq et al., 2020). The accumulation of these mutations disrupts the body’s homeostasis, leading to the transformation of normal cells into cancerous ones (Liu et al., 2015). For example, mutations in the tumor suppressor genes MAD4, TP53, DCRC, and APC, as well as oncogenes KRAS, HER2, and C-MYC, are important factors for GI tumors development (Baugh et al., 2018; Wang and Fakih, 2021; Lee et al., 2022). The second oncogenic pathway involves epigenetic changes like aberrant DNA methylation, microRNA (miRNA) and noncoding RNA deregulation, and altered histone modifications (Grady, 2021). These alterations are present in virtually all cases of GI tumors (Okugawa et al., 2015). In addition, factors like cell proliferation, apoptosis, genetics, obesity, alcohol consumption, and *Helicobacter pylori* infection, have been implicated in the development of GI tumors (Narasimhan et al., 2020; Zhu and Li, 2023).

In the past half century, oncology treatment has evolved from chemotherapy to targeted therapy and immunotherapy (Liu and Langer, 2022). Recently, gene therapy has seen rapid development with various gene therapy drugs being used in clinical practice (Naldini, 2015). Gene therapy involves the introduction of exogenous normal genes into specific cells in order to correct or mitigate diseases resulting from genetic defects and abnormalities, with the ultimate goal of achieving therapeutic outcomes (Giamas, 2020). Gene editing technology is an important tool to study gene function. Among various gene editing techniques, the clustered regularly interspaced short palindromic repeats and associated protein 9 (CRISPR/Cas9) system, characterized by its superior efficiency and precision, is commonly preferred (Karimian et al., 2019). In 2015, scientists used CRISPR/Cas9 to create a mutation model in human intestinal organoids to study intestinal tumors for the first time (Matano et al., 2015). However, patient survival was not improved due to tumor heterogeneity and metastasis, highlighting the need for new treatment models (Zhao et al., 2022).

Organoid models, which are three-dimensional (3D) clusters originating from tumor tissue or tumor-specific stem cells, effectively replicate tumor characteristics and cellular diversity *in vivo* (Xu et al., 2022). These models have demonstrated significant advantages in elucidating the underlying mechanisms of tumorigenesis, progression, metastasis, and drug resistance, thereby offering valuable theoretical insights and practical tools for advancing precision medicine (Yoshida, 2020). In this review, we discuss the different *in vitro* models in current tumor research, the derivation of cells in patient-derived organoids (PDOs) models, and the application of PDO model technology in GI cancers, particularly the applications in combination with CRISPR/Cas9 gene editing technology, drug screening, drug development, personalized medicine, and tumor microenvironment simulation. Furthermore, we also highlight the existing constraints and obstacles that necessitate resolution for the progression of human-derived GI cancer organoids.

## 2 Models for tumor research

Tumor research relies on tumor models, which to some extent reflect the biological characteristics of human tumors and provide a fundamental guarantee for tumor mechanistic research and clinical transformation applications (Wang et al., 2022a). At present, commonly used tumor models in tumor research include cell lines, patient-derived xenograft (PDX) models, patient-derived tumor-like cell clusters (PTCs) models, organoids models, *etc.* They play important roles in the mechanisms of tumorigenesis, drug screening, drug development, and translation to clinical applications.

### 2.1 Cell line model

Cell line model is the most commonly used models in tumor research. A tumor cell line is a kind of immortal cell model established by using patient tumor tissues through a planar culture system, which has the advantages of easy construction, a short culture cycle, a high survival rate, and high throughput drug screening (Morgan et al., 2017). In the 1974s, studies established the first human immortal cell line, the HeLa cervical cancer cell line. With the continuous improvement of cytogenetics, 2D culture systems and other technologies, cell lines have been widely used as the most convenient and efficient tumor models (Nelson-Rees et al., 1974). With the research deepening, the shortcomings of cell line model have been gradually revealed. Under 2D culture conditions, cells are unable to maintain the genetic phenotype and genetic heterogeneity of the original tumor cells due to the lack of an immune microenvironment and vascular network system and are unable to recapitulate the morphology and function of the original tumor tissues (Nelson-Rees et al., 1974). In addition, cell line cells are homogeneous cells that have been stably passaged *in vitro*, with poor heterogeneity, making it impossible to simulate the types and interactions of tumor cells *in vivo*. As a result, many drugs that worked well in early cell line trials have proved ineffective in subsequent clinical trials (Habanjar et al., 2021).

### 2.2 PDX model

In recent years, researchers have been committed to developing experimental models that can approximately and realistically reflect the tumor characteristics of patients *in vivo*. Successfully, two of the most representative models, the PDX model and the PDOs model, have been constructed (Lannagan et al., 2021). The PDX model refers to xenografts obtained by directly transplanting tumor tissues derived from patients into an immunodeficient animal. Since transplantation directly using tumor tissue preserves the tumor cells and their cellular matrix to a large extent, PDX preserves the heterogeneity of the original tumor and the interaction between the tumor and the surrounding matrix. However, PDX cannot fully capture the genetic heterogeneity in the original tumor (Kemper et al., 2015; Morgan et al., 2017). Morgan et al. examined the number of mutations in the PDX model of patient with primary non-small cell lung cancer and found that only 43% of mutations were detected, whereas four mutations that were not present in the original tumor were found early in the PDX process, suggesting that clonal selection and mutation of tumor tissues may occur during the early stages of implantation in mice (Morgan et al., 2017). Moreover, during PDX model generation, mouse stroma gradually replaces human stromal components. Since the human tumor microenvironment cannot be replicated exactly in mice, the interaction between human tumor tissues and the mouse stromal microenvironment affects tumor secretion signaling, which is detrimental to the study of stroma-directed therapies (Yoshida, 2020). In addition to the above drawbacks, the PDX model has several other limitations, such as being cost-effective, time-consuming, and inadequate for high-flow drug screening (Praharaj et al., 2018). Although PDX has been extensively utilized in several fields, including preclinical drug evaluation and biomarker identification, it is still not the most ideal preclinical model *in vitro* (Wilding and Bodmer, 2014).

### 2.3 PTC model

PTCs arise from the self-assembly and proliferation of immune cells, fibroblast, and primary epithelial that physically and functionally mimic the original tumors. The PTC model was originally proposed as an *in vitro* tumor model by the Xi team in 2020 (Yin et al., 2020). The model emphasizes strategies for the maintenance and expansion of primary tumor cells under matrigel-free conditions. Suspended tumor cell clusters are established using patient-derived specimens (e.g., tumor tissue, puncture specimens, ascites specimens, *etc.*) through the use of specially optimized media and low-adherence culture plates. The PTC model has shown potential for clinical applications in the fields of tumor drug resistance, drug development, and precision medicine due to the short culture cycle, low requirement for specimen sources, and high throughput drug screening. Yin et al. established *in vitro* PTC models from patients with GC and CRC for preclinical drug testing (Yin et al., 2020). PTC tests of 59 patients with gastric, colorectal, or breast cancers revealed an overall accuracy of 93% in predicting their clinical outcomes. Additionally, Liu et al. generated PTC models from lung cancer patients that structurally and functionally recapitulated the original tumor features and exhibited a high degree of morphological similarities to surgically resected samples, which can be used to assess the efficacy of cisplatin-based chemotherapy (Liu et al., 2023a). Zhang et al. evaluated the responses of PTCs to specific drugs or combinations of drugs (Zhang et al., 2023a). In a study by Peng et al., PTC models were conducted to investigate PD-L1 mRNA expression in extracellular vesicles, and data showed that PTCs are significantly heterogeneous (Peng et al., 2023). However, the PTC model has just been proposed in recent years, later in time than the PDO model. Unlike the 3D matrix-embedded culture method, the tumor cell clusters obtained by the PTC technique cannot be used for gene editing and immortalization in culture. PTC models generated for clinical studies are still rare, so more reports on PTC models are needed for public acceptance.

### 2.4 Organoid model

Organoids have the ability to self-renewal and self-assembly, retain the physiological structure and function of the original tissue, and proliferate indefinitely under *in vitro* culture conditions (Vlachogiannis et al., 2018). In 2009, Sato et al. introduced organoid technology in the experiment for the first time and successfully cultured mouse small intestine cells dependent on stem cell culture (Sato et al., 2009). Subsequently, in 2013, Huch et al. generated a 3D organoid model from mouse pancreatic tissue (Huch et al., 2013). The successful attempts and applications of organoids in normal tissues have made organoid culture technology develop rapidly in the field of tumor research. In 2015, Clevers and Tuveson LABS collaborated to build organoid models of human and mouse ductal pancreatic cancer (Koikawa et al., 2021). These tumor organoids have a critical trait in that they can partially or totally replicate the heterogeneity of the original tumor, making them ideal for tumor research. Compared to cancer cell lines, organoid models reflect the phenotypic and genetic characteristics of the original tumor with higher fidelity (Li et al., 2023). At the same time, the culture success rate and culture cycle were significantly improved compared with PDX, suggesting that organoids may be a more effective and ideal model for detecting novel anticancer drugs (Neal et al., 2018). Additionally, the immortality characteristics non-possessed in the PTC model showed the organoid model has a broader application scenario. Table 1 summarizes the advantages and disadvantages of the cell lines, PDX, PTC, and organoid models.

**TABLE 1:** Comparison of tumor models.

Model	Cell line	PDX	PDO	PTC
Cost	**+**	**+++**	**++**	**++**
Time consumption	**+**	**+++**	**++**	**++**
Success rate	**+++**	**+**	**++**	**++**
Genetic modification	**+++**	**+**	**++**	**-**
Genetic background retention	**-**	**+++**	**+++**	**+++**
Tumor heterogeneity	**-**	**++**	**+**	**+**
Drug efficacy prediction	**-**	**++**	**++**	**++**
High-throughput drug testing	**+++**	**-**	**++**	**++**
Personalized therapy	**-**	**++**	**+++**	**+++**
TME interaction research	**-**	**+++**	**++**	**++**
lmmunotherapy evaluation	**-**	**+++**	**++**	**++**
Biobanking	**-**	**-**	**+++**	**-**

PDX: patient-derived xenograft, PDO: patient-derived organoid, TME: tumor microenvironment, +: low correlation, ++: medium correlation, +++: high correlation, -: not suitable.

## 3 GI tumor Organoid derivation and culture

**FIGURE 1:**
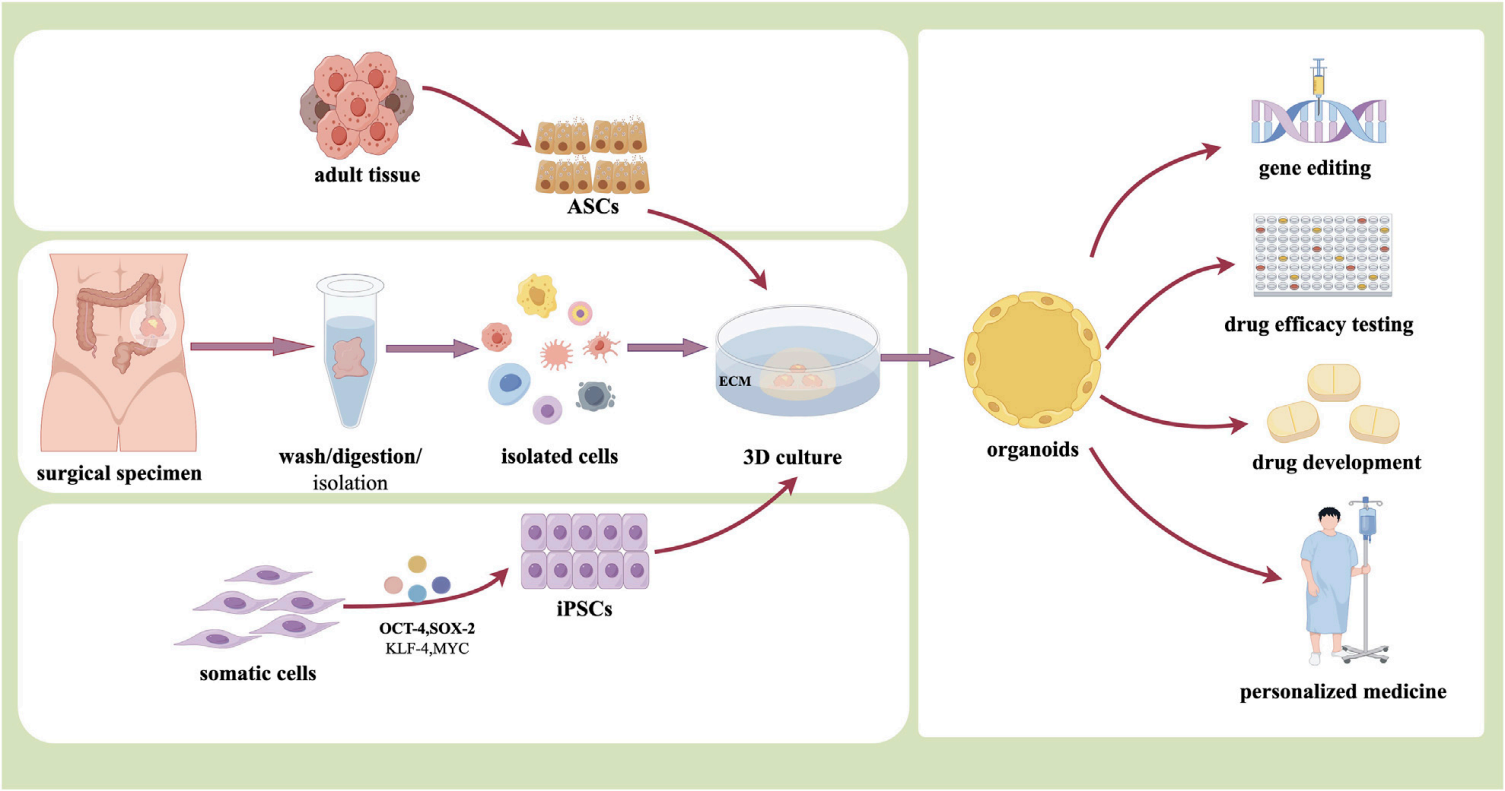
The organoid derivation and application (By Figdraw).

Organoids refer to cells obtained by surgical resection of tumor specimens, or biopsy tissues, or liquid biopsy samples cultured into 3D multicellular clusters with proliferation and spatial self-organization ability under specific culture conditions *in vitro*, which are also described as tiny tumors preserved in Petri-dishes (Artegiani and Clevers, 2018). Currently, the Matrigel is commonly used as a stereoscopic scaffold to support cell-cell and cell-matrix interactions in organoid culture (Onfroy-Roy et al., 2020; Xu et al., 2021). Matrigel is an ECM rich in laminin secreted by Engelbreth-Holm-Swarm tumor system, which contains a variety of growth factors and can regulate cell growth and metabolism (Kozlowski et al., 2021; Zhou et al., 2023). In the organoid culture system, the components that also need to be added mainly include activators, inhibitors and other specific cytokines such as epidermal growth factor and fibroblast growth factor, to ensure cell growth and differentiation (Almeqdadi et al., 2019; Seidlitz et al., 2019) (Figure 1).

### 3.1 Organoids from human pluripotent stem cells (PSCs)

Organoids can be differentiated from human PSCs or progenitor cells, which reserve certain functions and have similar cell types and tissue structures as internal organs (Reza et al., 2021). PSCs, including embryonic stem cells (ESCs) and induced PSCs (iPSCs), have been widely used in biomedical and biological sciences due to their potential to differentiate into all types of cells in the body (Yamanaka, 2020). The first pluripotent cell line was isolated and established from mouse blastocysts and reported in 1981 (Martin, 1981). After nearly 20 years of exploration, human ESCs were established by Thomson et al., in 1998 (Thomson et al., 1998). In 2006, Takahashi and Yamanaka reprogrammed mouse fibroblasts into PSCs by inserting four transcription factors encoding genes Oct4, Sox2, Klf4, and c-Myc. These cells, also known as iPSCs, have an intrinsic potential to develop into cells of all three germ layers *in vivo*, similar to those derived from ESCs (Takahashi and Yamanaka, 2006). Subsequently, human iPSC (hiPSC) technology was first introduced in 2007 and is now broadly used to generate human “dish disease” models. This hiPSC technology allows for personalized disease modeling and will be an important component of precision medicine (Takahashi et al., 2007).

Currently, hiPSC technology has been able to form intestinal, kidney, brain tissue, retina, liver, and other organoids *in vitro* (Taguchi and Nishinakamura, 2017; Workman et al., 2017; Nieto-Estévez and Hsieh, 2020; Zahmatkesh et al., 2021; Yamasaki et al., 2022). In 2011, Kyle W et al. developed a method to differentiate human embryos and PSC into intestinal organoids *in vitro*. In this study, hiPSC was first exposed to Activin A for definitive endoderm induction, then exposed to FGF3/Wnt4A to produce midgut/hindgut spheroids for 4 days, and then the spheroids were allowed to expand into intestinal tissues for 3–14 days (Spence et al., 2011). With the rapid development of regenerative medicine and precision therapy, it has been found that iPSC-derived organoids are more suitable for the study of reproducing the development process and internal changes of tissues or organs in time and space in human developmental biology. At the same time, the controversy surrounding its clinical application is highlighted by its tumorigenicity and heterogeneity.

### 3.2 Organoids from adult stem cells (ASCs)

Tissue-specific stem cells, also known as ASCs, exist in adult somatic tissues and contribute to tissue homeostasis and repair. ASCs have the capacity to self-renew and differentiate into specific cell types. The organoids formed by ASCs can well simulate the process of self-renewal or regeneration of tissues and organs after damage. Toshiro Sato and his colleagues established the first intestinal organoid model derived from ASCs in 2009 (Sato et al., 2009). The model entailed embedding mouse intestinal crypt leucine-rich repeat-containing G-protein-coupled receptor five-positive (LGR5^+^) cells with ECM and culturing with the necessary growth factors, including R-spondin-1, EGF, and BMP inhibitor Noggin. These organoids largely replicate the tissue architecture *in vivo* and contain relatively complete complements of stem cells, progenitor cells, and terminally differentiated cell types (Sato et al., 2009). Then, by changing the combination of growth factors and cell separation procedures, researchers can rapidly alter the organoid culture system to produce a variety of normal and cancerous human organoids, such as the colon, stomach, and liver (McCracken et al., 2014; Huch et al., 2015; Pleguezuelos-Manzano et al., 2020).

Compared with PSC-based organoids, ASC-based organoids are directly obtained from regenerated adult tissues, showing the advantages of simpler operation and shorter culture time. The maturity of ASC-derived organoids is closer to that of the source tissues, so ASC-derived organoids provide a better model for adult tissue repair (Tang et al., 2022). Unfortunately, ASC-derived organoids are not able to obtain **s**tem cell-based tissues from adult organs such as the brain, heart, and islet. However, either ASC-derived organoids or PSC-derived organoids can maintain the genetic stability of source organs or tissues while expanding *in vitro* for long periods, making them an ideal choice for cell expansion and promoting their potential applications in new therapeutic strategies (Kimura et al., 2022).

PDOs, especially tumor-derived organoids, are a special type of ASC-based organoids. For PDOs, samples were mainly obtained from surgery, biopsy, ascites, or other means. The samples are mechanically minced, enzymatically digested, and erythrocytically lysed to obtain cell suspensions, which are then coated with special culture media and matrix gel to form 3D structures. In 2015, Wetering M et al. established tumor tissue-derived organoids in 20 patients with CRC. The tumor organoids model is suitable for high throughput drug screening and can detect gene-drug association (van de Wetering et al., 2015). Another study by Nuciforo et al. showed that hepatocellular carcinoma organoids could be successfully obtained by digesting tumor tissues obtained from needle biopsies of hepatocellular carcinoma with type IV collagenase and inoculating them with type II basement membrane extracts. The histomorphological and genetic characteristics the hepatocellular carcinoma organoids also retained those of the original tumor tissues (Nuciforo et al., 2018). Currently, the method of directly obtaining tumor cells from patients to establish tumor organoids is relatively mature and most widely used, through which a variety of PDO models such as CRC, cholangiocarcinoma (Saito et al., 2019), and squamous cell carcinoma of the head and neck have been successfully established (van de Wetering et al., 2015; Driehuis et al., 2019).

## 4 Application of organoids in GI tumors

### 4.1 Organoids and CRISPR/Cas9 technology

**FIGURE 2:**
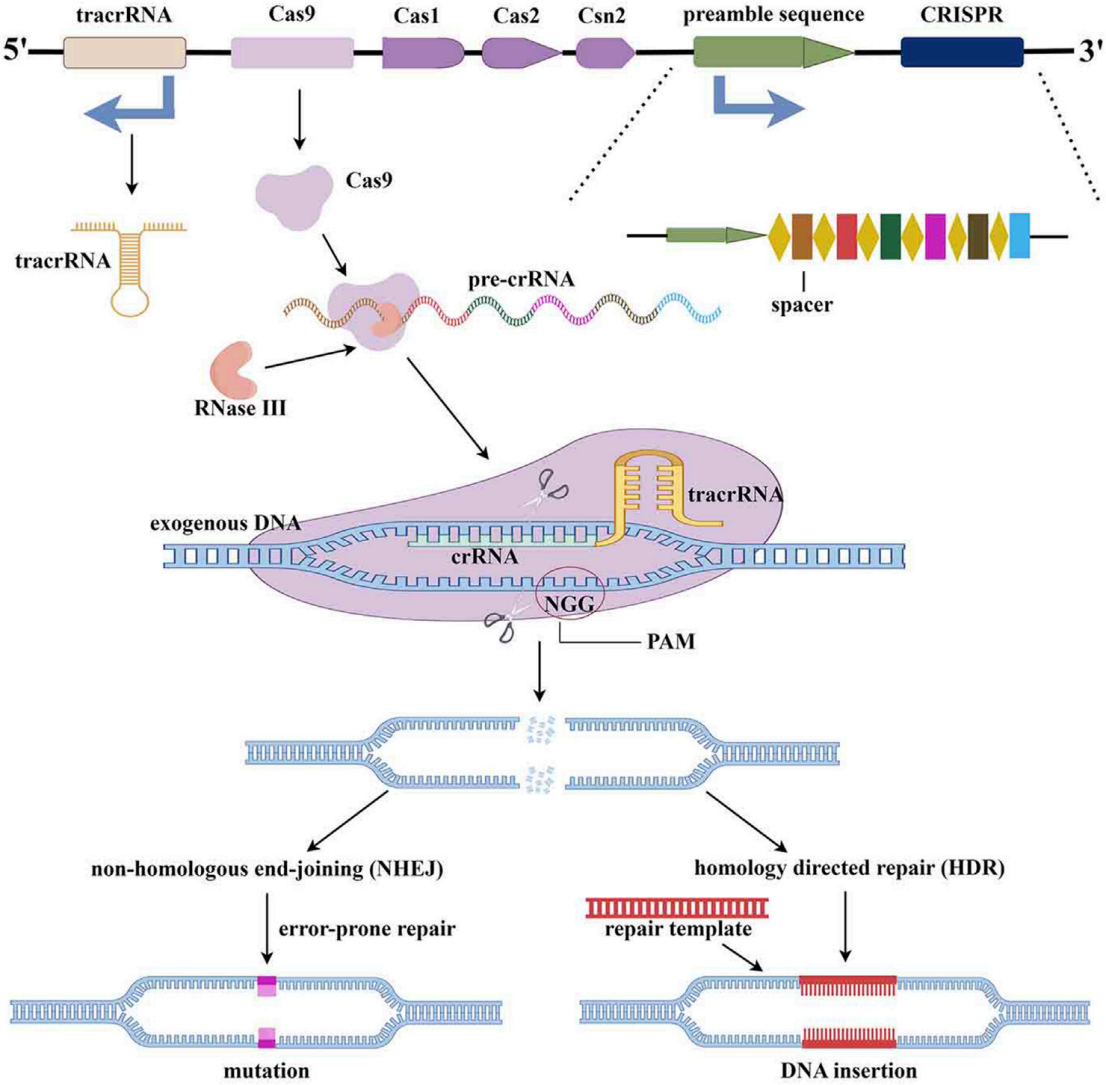
The mechanism of CRISPR/Cas9 gene editing technology (By Figdraw).

The CRISPR/Cas9 system is an adaptive immune defense developed by bacteria and archaea in the long-term evolution process to resist the invasion of viruses and exogenous DNA (Janik et al., 2020). CRISPR was first discovered and described in *Escherichia coli* bacteria DNA by Ishino et al. at Osaka University, Japan, in 1987. Subsequently, its powerful shearing and editing capabilities were definitively investigated in 2010 (Ishino et al., 1987). CRISPR/Cas9 consists primarily of the enzyme Cas9 and small guide RNA (sgRNA). SgRNA is a hairpin structure formed by complementary pairing of portions of crRNA and tracrRNA, and forms a complex with the Cas9 enzyme upon recruitment of sgRNA (Dominguez et al., 2016). Through the structure of Proto Spacer Adjacency Motif (PAM), the complex of CAS9 and sgRNA identifies and binds to the endogenous target genome. After the double-stranded DNA is unraveled, sgRNA will hybridize to complementary strand DNA, and then Cas9 enzymes cleave the complementary and non-complementary strands to produce flat ends. Finally, the target DNA of the double-strand break is destroyed during repair, leading to DNA silencing (Figure 2) (Dominguez et al., 2016; Janik et al., 2020).

The CRISPR/Cas9 system has been widely used in basic medicine, translational medicine, and other fields due to its high efficiency and operability in gene editing. Here, we only focus on the study of target gene mutations introduced by CRISPR/Cas9 technology in the organoid platform and their xenograft or not, while other studies such as target gene mutations introduced in cells and xenografts have not been adopted (Table 2). As one of the sharpest and most promising gene editing tools, the CRISPR/Cas9 system has been applied to gene knockout in many types of organoid platforms. In CRC, driver genes such as DACH1 (Hu et al., 2020), LARGE2 (Dietinger et al., 2020), and KIT (Küçükköse et al., 2022) were knocked out by using CRIPSR/CAS9 gene editing technology to investigate the promoting effects of intestinal stemness, cell-matrix adhesion, and phenotype switch in carcinomatosis, respectively. In order to explore the epigenetic contribution in GC, the histone lysine demethylase-1A (KDM1A) gene was knocked out in PDOs, which in turn induced inhibition of Wnt signaling and a strong cell cycle arrest (Mircetic et al., 2023). For the drug susceptibility test, Verissimo CS et al. evaluated the effect of mutant KRAS on drug response and found that dual inhibition of the EGFR-MEK-ERK pathway in RAS mutant organoids induced a transient cell-cycle arrest rather than cell death (Verissimo et al., 2016). In 2021, Boos SL et al. further demonstrated that metastatic CRC PDOs introduced by KRASG12D are an ideal platform to model chemotherapy tolerance *ex vivo* and AURKA was found to be a therapeutic target in liver metastatic CRC (Boos et al., 2022). Besides, the SMAD4 (Pfohl et al., 2022) mutation and FGFR1 and OXTR (Tung et al., 2021) mutations were knocked out using CRIPSR/CAS9 technology in colon cancer PDOs for reversing drug resistance. Using an organoid platform based on CRISPR/Cas9 knockout technology, Seino T et al. found that GATA6 expression regulated Wnt niche independence, which was mainly acquired non-genetically through mutations of driver genes (KRASG12V, CDKN2A, TP53, and SMAD4) during pancreas tumorigenesis (Seino et al., 2018). In another report by Hirt CK et al., ARID1A and BRCA were knocked out in PDAC organoid by CRISPR/Cas9 technology for identifying drug repurposing candidates. Researchers have successfully screened 26 out of 1,172 FDA-approved compounds, showing effective killing of PDAC organoids, including 19 chemotherapy drugs currently approved for other cancer types (Hirt et al., 2022). As we all know, gene knock in of CRISPR/Cas9 technology is also another commonly used gene editing technology in tumor research. Cortina C et al. devised a strategy based on engineered human CRC organoids that carry EGFP and lineage-tracing cassettes knocked in the LGR5 locus to study cancer stem cells in human tumors (Cortina et al., 2017). This strategy described herein may have broad applications to study cell heterogeneity in human tumors. Overall, the combination of the organoid platform with the CRISPR/Cas9 system conquers the drawbacks of conventional cell line culture and provides a more accurate representation of the biological behavior of tumor cells, making this a potent platform for tumor research.

**TABLE 2:** Application of CRISPR/Cas9 technology in PDOs.

	DATE	Target	Type of gene editing	Organoid	Xenotransplantation of organoids	Function	Ref
1	2020	DACH1	knock out	CRC organoids	—	the importance of DACH1 for CRC organoid formation and stemness	Hu et al. (2020)
2	2020	LARGE2	knock out	human colon tumor organoid	—	silenced LARGE2 compromised O-glycosylation of α-DG in CRC	Dietinger et al. (2020)
3	2022	KIT	knock out	CRC Organoid	NSG	caused a partial mesenchymal-to-epithelial phenotype switch and a strong reduction of intra-tumor stromal content	Küçükköse et al. (2022)
4	2023	KDM1A-KO	Epigenetic library for negative select screen	GC PDOs	—	assess the contribution of epigenetic regulators to gastric cancer	Mircetic et al. (2023)
5	2016	KRAS^G12D^	knock out	patient-derived CRC organoid	—	evaluated RAS pathway inhibitors and drug combinations that are currently in clinical trial for RAS mutant cancers *in vitro*	Verissimo et al. (2016)
6	2021	KRAS^G12D^	knock out	CRC organoids	—	specific effect of KRASG12D acquisition in drug-tolerant organoids	Boos et al. (2022)
7	2022	SMAD4^R361H^	knock out	CRC organoids	—	drug screening and reverses MEK-inhibitor resistance	Pfohl et al. (2022)
8	2019	FGFR1 or OXTR	knockout	colon cancer organoids	—	help overcome oxaliplatin resistance	Tung et al. (2021)
9	2018	GATA6, KCTS	knock out	PDAC organoids	NOD.Cg-*Prkdc* ^scid^Il2rg^*tm1Sug*^/Jic (NOG)	demonstrated the stepwise tumorigenesis of PDAC with progressive acquisition of niche independency	Seino et al. (2018)
10	2022	ARID1A, BRCA2	knock out	human PDAC organoid	—	drug screen for identifying candidates	Hirt et al. (2022)
11	2017	LGR5‐EGFP and KI67‐TagRFP2	knock in	CRC organoids	NOD/SCID female mice	integrate reporter cassettes at desired marker genes	Cortina et al. (2017)

PDOs: patient-derived organoids, CRC: colorectal cancer, GC: gastric cancer, PDAC: pancreatic ductal adenocarcinoma.

### 4.2 Organoids for drug screening

The research on tumor organoids in drug screening mainly focuses on the establishment of tumor organoid models and tumor organoid biobanks to evaluate the anti-tumor activity of drugs, and predict drug targets and drug sensitivities (Driehuis et al., 2020). Due to their suitability for high-throughput screening, PDOs are an efficient platform for tumor drug screening (Wang and Jeon, 2022). Compared with animal models and two-dimensional cell models, tumor organoids not only have a higher success rate, a shorter culture cycle, and more facility for large-scale culture and drug screening through pore plates, but they also retain the heterogeneity and physiological and pathological changes of tumors in patients. Therefore, tumor organoids are more suitable for evaluating the treatment effects, toxicity, and side effects of drugs *in vivo* (Mittal et al., 2019), and thus for improving the efficiency of drug development and the success rate of drug clinical trials.

#### 4.2.1 CRC PDOs

Most CRCs are aggressive and lack effective strategies for selecting appropriate anticancer regimens. CRC PDOs have emerged as preclinical platforms for modeling clinical responses to drug screening. The two most common strategies for drug screening are targeted drug discovery (TDD) and phenotype drug discovery (PDD). The latter does not depend on an understanding of the target and mechanism. It can also be divided into drug screening based on a compound library or drug screening based on an antibody library. In recent years, fedratinib (Mao et al., 2023), vinorelbin (Mertens et al., 2023), metformin (Luo et al., 2023), *etc.*, with special anti-cancer effects on CRC have been screened from the compound library. Besides, Soon-Chan Kim et al. demonstrated a platform of 12 sets of PDOs and cell lines isolated from multiple regions of single tumors from 12 patients that were used to clarify heterogeneous drug responses using a clinically relevant 24-compound library (Kim et al., 2022). MCLA-158, a bAb that specifically triggers epidermal growth factor receptor degradation in LGR5^+^ cancer stem cells but shows minimal toxicity toward healthy LGR5+ colon stem cells, was tested in a large-scale functional screen. Briefly, using the CRC organoid platform, MCLA-158 was identified as a therapeutic EGFR × Lgr5 bispecific antibody from 500 effective bAbs against epithelial tumors by Herpers B et al. (Herpers et al., 2022). Interestingly, De Angelis ML et al. generated organoids from CTCs isolated from an orthotopic CRC xenograft model and found that CTC-derived organoids (CTCDOs) have an increased sensitivity to YM155, a drug targeting Survivin, and to the HSP90 inhibitor AUY922 (Luminespib) (De Angelis et al., 2022). In contrast, a prospective experimental treatment of CRC patients based on organoid drug responses revealed that drugs that have been observed to be therapeutic on organoid models may not achieve the expected consistent clinical responses in patients (Ooft et al., 2021). However, the authors of this research also point out that limited drugs and limited number of patients do not allow firm conclusions on whether organoid *in vitro* sensitivity predicts clinical response *in vivo*.

#### 4.2.2 GC PDOs

GC PDOs are widely heralded as a drug-screening platform to develop new anti-cancer therapies. Yan et al. established a primary GC organoids (GCO) biobank, with which 37 anticancer drugs were screened (Yan et al., 2018). Drugs recently approved or in clinical trials, such as Napabucasin, Abemaciclib, and the ATR inhibitor VE-822, were confirmed to be effective in large-scale screening. In other words, this GCO biobank, with linked genomic data, provided a useful resource for studying both cancer cell biology and precision cancer therapy. Li et al. established a 3D organoid culture derived from malignant ascites (MA) of GC for disease modeling and drug screening. Eleven MAPDOs were produced from MA tumor cells in GC cells (Li et al., 2019). In another study, different responses to 5-fluorouracil, oxaliplatin, docetaxel, and irinotecan were observed in sig-ring cell carcinoma (SRCC) and non-SRCC GC organoids, predicting a potential strategy for drug response testing (Puccini et al., 2022). SRCC is specific and heterogeneous in phenotype and drug resistance. Systemic therapy is often ineffective for patients with SRCC, so predictive biomarkers are urgently needed to guide treatment.

#### 4.2.3 Other PDOs

The PDOs platform has also been wildly established for drug-screening in other GI tumors, such as pancreatic ductal adenocarcinoma, HCC, *etc.* For example, Li et al. generated an organoid-based tissue platform using patient tissues from pancreatic ductal adenocarcinoma and assessed the cellular response to standard-of-care chemotherapeutic compounds, demonstrating their usability for drug screening (Li et al., 2022). Recently, Zou et al. performed the drug sensitivity test of Atezolizumab on microchips based on PDO, MSC‐PDO‐PBMC, CAF‐PDO‐PBMC, and PDO‐PBMC models (Zou et al., 2023). The later three models showed a more sensitive response, indicating that MSC (CAF)-PDO-PBMC models mimicking the original tumor microenvironment (TME) were more suitable than conventional PDO models for *in vitro* tests of immunotherapeutic drugs.

### 4.3 PDOs for anti-tumor drug development

Although a large number of innovative anticancer drug projects are declared every year, there are few ideal drugs that eventually enter clinical application. The reason is that in the pre-clinical research stage of drug development, there is no suitable drug development model to simulate the *in vivo* environment to evaluate the efficacy of drugs, resulting in most drugs showing good results in basic research, but in clinical trials, satisfactory results cannot be achieved. The organoid model can not only enable cells to be cultured in 3D, but also better evaluate the therapeutic effect of drugs by simulating the growth environment *in vivo*. It has been widely used in GI tumor research.

#### 4.3.1 PDOs is an important *in vitro* model for mechanism research and targeted drug development

PDOs have been established for many types of cancer and have become a promising tool for personalized oncology research. These models can completely reproduce the pedigree of tumor development and can be used for large-scale analysis of therapeutic responses, that is, drug typing, biomarker discovery, and tumor-related pathway research (Piro et al., 2021). In CRC, Roulis M et al. described rare pericryptal Ptgs2-expressing fibroblasts exerting paracrine control over tumor-initiating stem cells via the druggable PGE2-Ptger4-Yap signaling axis and considered that the initiation of CRC correlates with the mesenchymal niche (Roulis et al., 2020). Besides, the signaling axis of CSN6-TRIM21 was reported to promote tumor stemness during tumorigenesis (Qin et al., 2020). TFEB-PGD was considered another signaling axis activated by ATP13A2 to promote tumor growth (Zhang et al., 2023b). In GC, signaling axis of KDM1A-NDRG1 and NF-κB-PIEZO1-YAP1-CTGF might serve as potential therapeutic options to block tumorigenesis (Chen et al., 2023; Mircetic et al., 2023). Besides, Chromosome eight open reading frame 76 (C8orf76) was for the first time identified as a novel therapeutic target for GC that directly binds to oncogenic lncRNA DUSP5P1 to induce its expression and activate MAPK signaling (Wang et al., 2019). Moreover, pyruvate kinase M2 isoform (PKM2) mediated by ARRB1 may be a promising therapeutic strategy in a subset of GC patients (Yu et al., 2022). Similarly, in hepatocarcinogenesis, Wong TL et al. found that CRAF methylation by PRMT6 regulates aerobic glycolysis-driven via ERK-dependent PKM2 nuclear relocalization and activation (Wong et al., 2020). In another ferroptosis study in hepatocellular carcinoma, WTAP was investigated as a key factor upregulating ATG5 in an m^6^A-YTHDC2-dependent manner and targeted inhibition of WTAP may be an innovative and effective treatment strategy for HCC (Li et al., 2024). Through a cohort study, the Zhou SL team evaluated broad differences among organoids with different BRAF variant subtypes in sensitivity to BRAF or MEK inhibitors, and suggested a precise treatment for patients with intrahepatic cholangiocarcinoma (ICC) (Xin et al., 2023).

#### 4.3.2 PDOs is also an important *in vitro* model for drug development

In the era of precision medicine, clinical medical staff and oncologists are eager to find more realistic, economical, and timely *in vitro* tumor models to assist drug development. The emergence of the PDO platform seems to solve the urgent problem. Recently, Kim N et al. demonstrated that fisetin, a natural plant flavonoid, significantly delayed tumor growth and inhibited vascular endothelial growth factor (VEGF) and epithelial cell adhesion molecule (EpCAM) via upregulation of AKAP12 in CRC (Kim et al., 2023). Besides, ATP13A2 and CaCO3@Cur@QTX125@HA nanoparticles were also reported as potential targets for CRC therapy (Zhang et al., 2023b; Hu et al., 2023). Moreover, MCLA-158, as mentioned above, exhibits potential therapeutic properties in epithelial tumors (Herpers et al., 2022). In two HCC studies, SHP099, a Src homology two domain-containing phosphatase two inhibitor, and desloratadine were reported as novel anticancer drugs (Leung et al., 2020; Tan et al., 2023). In another pancreatic cancer research, Krukovine was found to be an effective therapy in Oxaliplatin-resistant pancreatic cancer cells (Lee et al., 2023). In addition, NTRC 0652-0, a novel LCK selective tyrosine kinase inhibitor (Conboy et al., 2023), and Heat Shock Protein 90 were reported as novel inhibitors in cholangiocarcinoma (Lampis et al., 2018). These findings reveal that this PDO model is consistent with the original tumor, which has great potential for personalized cancer therapy and is a prerequisite for subsequent drug screening.

#### 4.3.3 PDO is also an important *in vitro* model for reversing drug resistance

Intrinsic and acquired resistance to therapeutic drugs occurs in virtually all types of cancer. For effective treatment, the development of new drugs or combination therapy plays an important role in eliminating extensive drug resistance. In CRC, it is reported that co-treatment with embedded proteasome inhibitors bortezomib and cisplatin in nanoparticles can improve efficacy and reduce the side effects caused by drug combination therapy (Shao et al., 2020). Over more, Mcl-1 inhibition and lnc-RP11-536 were reported as the critical contributors to reversing the resistance of regorafenib and oxaliplatin, respectively (Song et al., 2020; Li et al., 2021). Using the PDO model established from GC patients, BDNF was discovered as a therapy target to reverse anlotinib resistance, and MYOF was found to be a promising biomarker and therapeutic target for L-OHP-resistant patients (Harada et al., 2021; Jin et al., 2021). Additionally, in HCC, lysosomal protein transmembrane five and protein arginine N-methyltransferase 6 (PRMT6) were considered the critical contributors to sorafenib and lenvatinib resistance, separately (Pan et al., 2023; Li et al., 2024). For pancreatic cancer, Irbesartan overcomes gemcitabine resistance by suppressing stemness and iron metabolism via inhibition of the Hippo/YAP1/c-Jun axis. The combination of gemcitabine and mithramycin shows potential therapeutic efficacy in reversing the resistance of gemcitabine.

### 4.4 Clinical research and personalized treatment

Recently, the PDOs model has been applied in clinical trials for personalized drug selection. The utility of PDOs in large-scale prospective clinical cohorts contributes to deeply integrating molecular biological characteristics and treatment responses in cancer patients, reducing the time of the clinic-laboratory-clinic cycle, and construct the platform for personalized oncology therapy (Shankaran et al., 2021). 40 case GI tumor PDO-related clinical trials have been registered in the Clinical Trials (Table 3).

**TABLE 3:** Registered clinical studies of PDO in GI cancer.

Register ID	Status	Brief title	Estimated enrollment	Population	Intervention	Outcome	Study design	Nation	Registration date
NCT05401318	Recruiting	Tailoring Treatment in Colorectal Cancer (Target CRC)	40	Patients with suspected colorectal cancer referred into the program for standardized cancer diagnostic pathways at Akershus University Hospital	Tumor tissue sampling for organoid development	identification of chemotherapy or chemotherapy combinations with or without targeted therapies that induce immunotherapy efficacy in CRC	Observational	Norway	2022/3/28
NCT04996355	Recruiting	Organoids-on-a-chip for Colorectal Cancer and *in Vitro* Screening of Chemotherapeutic Drugs	52	patients with advanced colorectal cancer	Other: comprehensive treatment after MDT discussion	the accuracy, specificity and sensitivity of organoids-on-chip for drug sreening	Observational	China	2021/8/2
NCT06196554	Recruiting	Gastric Cancer Organoids in the Screening of Neoadjuvant Drugs	40	patients with locally advanced gastric cancer receiving neoadjuvant chemotherapy	Drug: Oxaliplatin	the proportion of inconsistencies between organoid drug screening results and actual clinical observation drug sensitivity	Observational	China	2023/12/1
NCT06100016	Recruiting	A Clinical Study Aims to Assess the Consistency of Clinical Efficacy in Colorectal Cancer Treatment and Drug Susceptibility Outcomes Using a Novel Drug Susceptibility Testing Method	105	patients with colorectal cancer in the First Affiliated Hospital of China Medical University	Not provided	evaluate the consistency of clinical efficacy in colorectal cancer treatment and drug susceptibility outcomes	Observational	China	2023/10/20
NCT06100003	Recruiting	A Clinical Study Aims to Assess the Consistency of Clinical Efficacy in Gastric Cancer Treatment and Drug Susceptibility Outcomes Using a Novel Drug Susceptibility Testing Method	104	patients with gastric cancer in the First Affiliated Hospital of China Medical University	Not provided	evaluate the consistency of clinical efficacy in gastric cancer treatment and drug susceptibility outcomes	Observational	China	2023/10/25
NCT03577808	NOT YET RECRUITING	Organoids in Predicting Chemoradiation Sensitivity on Rectal Cancer	80	Organoids in Predicting Chemoradiation Sensitivity on Rectal Cancer	Other: Biopsy	predict the clinical outcome in locally advanced rectal cancer patients underwent neoadjuvant chemoradiation	Observational	China	2018/7/5
NCT04906733	NOT YET RECRUITING	Cetuximab Sensitivity Correlation Between Patient-Derived Organoids and Clinical Response in Colon Cancer Patients	80	Patients with recurrent and metastatic colorectal cancer	Procedure: Tumor biopsy	to assess the consistency of organoid chemotherapy sensitivity and clinical chemotherapy efficacy	Observational	China	2021/5/28
NCT04497727	NOT RECRUITING	Gut Organoid Study	41	Hypertensive and normotensive patients who routinely undergo scheduled elective colonoscopy	Other: Gene Expression	compare basic properties of gut epithelia of hypertensive and normotensive reference subjects	Observational	United States	2020/8/4
NCT05832398	Recruiting	Precision Chemotherapy Based on Organoid Drug Sensitivity for Colorectal Cancer	186	Colorectal Cancer	Drug: FOLFOX, FOLFIRI or FOLFOXIRI regimens	to investigate whether chemotherapy guided by PDOs drug test can improve the outcomes of stage IV CRC.	Interventional	China	2023/4/27
NCT05294107	Recruiting	Intestinal Organoids (BIOÏDES)	90	Digestive System Diseases	Procedure: additional biopsies	generate a biocollection of 3D intestinal models from digestive biopsies with associated health data and to characterize them before using them for the screening of potential therapeutic molecules	Interventional	France	2022/3/24
NCT05038358	NOT YET RECRUITING	Tumor Immune Microenvironment Involvement in Colorectal Cancer Chemoresistance Mechanisms (CRC-ORGA 2)	60	colorectal adenocarcinoma, naive from neo-adjuvant chemotherapy, indication of surgical resection	Not provided	develop a model of tumoroids derived from patients with a colorectal tumors prior to any systemic anti cancer treatment	Observational	France	2021/9/9
NCT02874365	COMPLETED	Intestinal Stem Cells Characterization (BIODIGE)	110	Inflammatory Bowel Diseases	Inflammatory Bowel Diseases	investigate the morphological characteristics of organoids, the expression of genes and proteins of the Wnt/APC/beta-catenin pathway within both ISC.	Interventional	France	2016/8/22
NCT04371198	COMPLETED	Patient-Derived Organoids for Rectal Cancer	20	Rectum Cancer	Other: Biopsy	to determine the feasibility of establishing patient-derived organoids from pre-treatment rectal adenocarcinoma biopsies	Interventional	United States	2020/5/1
NCT03256266	Recruiting	Effect of Antigens or Therapeutic Agents on *in Vitro* Human Intestinal Organoids	375	Patients with patients with Food intolerances, Food allergy, intestinal disorders, glutensensitivity, healthy controls who underwent a gastroduodenoscopy or coloscopy for therapeutical intervention or screening	Not provided	the study evaluates the effect of nutrient antigens or therapeutic agents on human small intestinal organoids	Observational	Germany	2017/8/22
NCT05351398	NOT YET RECRUITING	The Clinical Efficacy of Drug Sensitive Neoadjuvant Chemotherapy Based on Organoid Versus Traditional Neoadjuvant Chemotherapy in Advanced Gastric Cancer	54	Patients with advanced gastric cancer whose tumor is located in the stomach and need neoadjuvant therapy before radical surgery	Drug: PDO group Drug: PDO group	access the safety and clinical value of the personalized neoadjuvant therapy based on patient-derived organoid drug sensitivity assay in advanced gastric cancer	Observational	China	2022/4/28
NCT05352165	NOT YET RECRUITING	The Clinical Efficacy of Drug Sensitive Neoadjuvant Chemotherapy Based on Organoid Versus Traditional Neoadjuvant Chemotherapy in Advanced Rectal Cancer	192	Neoadjuvant Therapy	Drug: standard long-term therapy	the organoid sample bank of more than 100 patients with locally advanced rectal cancer was completed	Interventional	Not provided	2022/4/28
NCT05183425	Recruiting	Patient-derived Organoids Predicts the Clinical Efficiency of Colorectal Liver Metastasis	60	All patients with histologically proven colorectal cancer with liver metastasis	Not provided	investigate the consistency of drug sensitivity for the matched primary and metastatic tumor in patients with liver metastasis	Observational	China	2022/1/10
NCT05203549	UNKNOWN STATUS	Consistency Between Treatment Responses in PDO Models and Clinical Outcomes in Gastric Cancer	250	Two hundred and fifty patients with gastric cancer who need to receive neoadjuvant therapy, conversion therapy or palliative chemotherapy are included in this study	Procedure: Tumor biopsy	to evaluate the consistency between treatment response in the PDO model and patient clinical outcomes	Observational	China	2022/1/24
NCT05425901	Recruiting	Preclinical Evaluation of Multimodal Therapeutic Strategies in Intestinal Irradiation and Inflammatory Bowel Disease From Organoids (INTRUST)	80	Radiation Enteritis Radiation Enteritis Inflammatory Bowel Diseases	Other: biopsy	to setup a screening tool by irradiating the organoids (step one) and then evaluate *in vitro* the regenerative activity of treatments dedicated to improve inflammatory bowel diseases and acute radiation enteritis	Interventional	France	2022/6/21
NCT05304741	Recruiting	The Culture of Advanced/​Recurrent/​Metastatic Colorectal Cancer Organoids and Drug Screening	30	Patients suffered from advanced/recurrent/metastatic colorectal cancer which was diagnosed as adenocarcinoma and unresectable	Not provided	organoids from patients with advanced/relapsed/metastatic colorectal cancer are established, and drug screening assays for organoids will be performed with chemotherapy and targeted agents and compared with clinical practice	Observational	China	2022/3/31
NCT05652348	Recruiting	Response Prediction of Hyperthermic Intraperitoneal Chemotherapy in Gastro- Intestinal Cancer (Hi-STEP1)	48	Eligible patients are recruited consecutively during of the initial surgical treatment planning	Other: Establishment of organoid cultures and *in vitro* sensitivity testing	organoid cultures from biopsies were established, various chemotherapeutic agents were tested on these tumor organoids, and genetic changes in tumor organoids were analyzed	Observational	Germany	2022/12/15
NCT06057298	Recruiting	Patient-tailored Hyperthermic Intraperitoneal Chemotherapy (HIPEC) for Colorectal Peritoneal Metastases	24	Peritoneal Metastases From Colorectal Cancer	Procedure: Patient-tailored HIPEC	to demonstrate that cytoreductive surgery and patient-tailored HIPEC will increase efficacy in controlling peritoneal disease	Interventional	Italy	2023/9/28
NCT05883683	NOT YET RECRUITING	Molecular Study and Precision Medicine for Colorectal Cancer (MSPM)	100	patients with advanced or recurrent colorectal cancer	Other: Molecular Profiling and drug testing in tumor organoids and PDXs	to identify clinical actionable targets and predict *in vivo* response of the tumor to targeted drugs by using PDOs/PDXs	Observational	China	2023/6/1
NCT05725200	Recruiting	Study to Investigate Outcome of Individualized Treatment in Patients With Metastatic Colorectal Cancer (EVIDENT)	40	Metastatic Colorectal Cancer	Drug: Alectinib、Cetuximab、Crizotinib	to investigate the effect and side effects of personalized cancer treatment in patients with metastatic colorectal cancer (bowel cancer)	Interventional/Phase 2	Norway	2023/2/13
NCT03283527	Recruiting	Organoid Based Response Prediction in Esophageal Cancer (RARESTEM/Org)	100	patients with curatively treatable and resectable esophageal cancer	Not provided	the steepness of the dose response survival curve in the organoids in relation to the pathologic response after resection in the clinical situation	Observational	Netherlands	2017/9/12
NCT05644743	NOT YET RECRUITING	Clinical Transformation of Organoid Model to Predict the Efficacy of GC in the Treatment of Intrahepatic Cholangiocarcinoma	40	patients were histologically or cytologically diagnosed with locally advanced inoperable radical resectable or metastatic intrahepatic cholangiocarcinoma	gemcitabine + cisplatin	build an organoid-based drug resistance prediction model	Observational	Not provided	2022/11/30
NCT04931394	Recruiting	Organoid-Guided Adjuvant Chemotherapy for Pancreatic Cancer	200	Pancreatic Cance	Other: Adjuvant chemotherapy guided by organoid drug sensitivity test	the consistency between the drug sensitivity test results and the treatment response of patients will be analyzed	Interventional/Phase 3	China	2021/6/18
NCT04931381	Recruiting	Organoid-Guided Chemotherapy for Advanced Pancreatic Cancer	100	Advanced Pancreatic Cancer	Other: Chemotherapy guided by organoid drug sensitivity test	explore the concordance between drug sensitivity test results and patients’ treatment response	Interventional/Phase 3	China	2021/6/18
NCT05196334	NOT RECRUITING	Pharmacotyping of Pancreatic Patient-derived Organoids	88	Patients with histopathological confirmation of pancreatic ductal adenocarcimona, ineligible for surgery, planned to start standard first line treatment	Other: No intervention	to use organoids cultured from diagnostic endoscopic ultrasound (EUS)-guided fine needle biopsy (FNB) samples from patients with PDAC for pharmacotyping	Observational	Denmark	2022/1/19
NCT05842187	Recruiting	*In Vitro* Organoid Drug Sensitivity-Guided Treatment for Metastatic Pancreatic and Gastric Cancer (ODYSSEY)	20	Pancreatic Cancer Gastric Cancer	Procedure: Biopsy of tumor tissue for organoid culture	to evaluate the consistency between *in vitro* tumor organoid drug sensitivity and the therapeutic efficacy of *in vivo* drug treatment	Interventional	China	2023/5/3
NCT05351983	Recruiting	Patient-derived Organoids Drug Screen in Pancreatic Cancer	50	Pancreas Cancer	Procedure: Surgical biopsy of tumoral tissue for organoid generation	help to speed up the implementation of organoid generation in the clinical routine for the choice of the best treatment of patients affected by pancreatic cancer	Interventional	Switzerland	2022/4/28
NCT05634694	Recruiting	Study on Consistency Evaluation for Drug Sensitivity of Patient-Derived Organoid Model From Cholangiocarcinoma Patients	40	Histologically confirmed cholangiocarcinoma patients who underwent radical resection and received post-operation adjuvant chemotherapy	Other: No interventions	evaluate the consistency and accuracy of patient-derived organoid model of cholangiocarcinoma to predict the clinical chemotherapeutic efficacy, as well as the possibility of guiding the adjuvant chemotherapy	Observational	China	2022/12/2
NCT04777604	NOT YET RECRUITING	Development of a Prediction Platform for Neoadjuvant Treatment and Prognosis in Pancreatic Cancer Using Organoid	300	patient who have been diagnosed with pancreatic cancer via EUS-FNA and EUS-FNB at Samsung Medical Center	Other: Organoid	to investigate the relationship between unique genomic mutations and responsiveness to anti-cancer drugs in patients with pancreatic cancer	Observational	Korea	2021/3/2
NCT03500068	UNKNOWN STATUS	Establishing Organoids From Metastatic Pancreatic Cancer Patients, the OPT-I Study. (OPT-1)	30	Carcinoma, Pancreatic Ductal	Carcinoma, Pancreatic Ductal	to develop a model system and infrastructure to individualize the treatment of patients with advanced pancreatic adenocarcinoma	Interventional	Netherlands	2018/4/17
NCT04736043	Recruiting	Development of a Prediction Platform for Adjuvant Treatment and Prognosis in Resected Pancreatic Cancer Using Organoid	300	All adult patients (>18 years) with (a suspicion of) advanced pancreatic adenocarcinoma	Other: Organoid	to investigate the relationship between unique genomic mutations and responsiveness to anti-cancer drugs in patients with pancreatic cancer	Observational	Korea	2021/2/3
NCT03544255	UNKNOWN STATUS	Drug Screening of Pancreatic Cancer Organoids Developed From EUS-FNA Guided Biopsy Tissues	50	Patients suspected to have pancreatic cancer who are going to receive EUS-FNA operations for diagnostic purposes	Procedure: Biopsy	establish organoid models from pancreatic cancer biopsies achieved via EUS-FNA	Observational	China	2018/6/1
NCT05932836	Recruiting	An Organoid-on-chips Technique Based on Biopsy Samples and Its Efficacy in Predicting the Response to HAI in HCC	165	Patiens who need to undergo biopsy for malignant tumors such as breast cancer, lung cancer, liver cancer, bile duct cancer or pancreatic cancer that have been clinically and/or pathologically diagnosed	Other: No interventions	to evaluate the predicting efficacy of the established organoid-on-chips system in HCC patients who undergo hepatic artery infusion (HAI) with mFOLFOX6	Observational	China	2023/7/6
NCT03990675	UNKNOWN STATUS	Evaluation and Comparison of the Growth Rate of Pancreatic Cancer Patient-derived Organoids	50	Patients with indication for EUS-guided FNA or FNB of a suspected pancreatic malignancy Exclusion Criteria	Procedure: FNA, FNB	evaluate and compare the growth rate of pancreatic cancer patient-derived organoids generated from matched fine needle Aspirations (FNA) and fine needle biopsies (FNBs)	Interventional	Germany	2019/6/19
NCT05571956	Recruiting	Establishment of Pancreas Cancer and Cancer-associated Fibroblast Using EUS-guided Biopsy Samples	50	Pancreas Adenocarcinoma	Other: Pancreatic ductal adenocarcinoma organoids and cancer-associated fibroblasts	simultaneously establish the patient-derived PDA organoids as well as CAFs using EUS-FNB samples	Interventional	Korea	2022/10/7
NCT02436564	UNKNOWN STATUS	*In Vitro* Models of Liver and Pancreatic Cancer	75	Patients who are undergoing surgical resenctions of liver, biliary or pancreas cancers that are able to give informed consent	Genetic: Organoid *in vitro* culture	develop an *in vitro* model of cancer for laboratory study using liver, biliary and pancreatic cancer tissue	Observational	United Kingdom	2015/5/7

PDOs: patient-derived organoids, GI: gastrointestinal, CRC: colorectal cancer, HIPEC: hyperthermic intraperitoneal chemotherapy, PDAC: pancreatic ductal adenocarcinoma.

### 4.5 Organoids and tumor microenvironment

The susceptibility of cancer to inflammation and immune deficiency demonstrates the critical role of immunity in tumorigenesis (Hibino et al., 2021). Cellular and drug immunotherapy can exert anti-cancer effects by enhancing the body’s immune monitoring function and local regulation of the tumor immune microenvironment, promising that the TME is a hot spot in the field of tumor research (Liu et al., 2023b). As we all know, the TME consists of immune cells, cancer-related fibroblasts (CAFs), interstitial cells, and ECM (Bejarano et al., 2021). The components in TME can maintain the differentiation activity of cancer stem cells and affect the occurrence, progress, metastasis, and drug resistance in tumorigenesis. At present, most tumor organoid models only contain tumor cells and lack TME components (Idris et al., 2021). Therefore, some research teams have constructed a co-culture system to explore the interaction between TME and PDOs (Mackenzie et al., 2022).

#### 4.5.1 Co-culture system of CAFs and PDOs

CAFs are one of the main components of TME, which can secrete stimulating signals to support tumor development, suppress immunity, and promote drug resistance (Mao et al., 2021). Recently, organoid models of gastrointestinal tumors co-cultured with CAFs have been continuously established and developed. CRC PDO-CAF models developed by Luo et al., demonstrated that CAF can maintain the proliferation of CRC PDO culture in hydrogels even without the addition of traditional growth factors with which CRC-PDO can be cultured (Luo et al., 2021). In addition, the immune response-related pathways that exist in patient tissues but are missed in the non-co-culture model are reactivated. To compare the effects of different fibroblasts on PDO, primary fibroblasts from tumor adjacent tissues and CAFs were co-cultured with CRC organoids, respectively. The co-cultured organoids revealed greater heterogeneity in tumor cells than monocultured organoids, and crosstalk between tumor cells and fibroblasts leads to some imbalance in biological pathways (Atanasova et al., 2023). Similarly, Strating et al. co-cultured colon cancer organoids and CAFs in a highly standardized serum-free medium and optimized the ECM to investigate the interaction between cancer cells and CAFs and their effect on immune-related cytokines and T cell proliferation (Strating et al., 2023). In addition, Dang et al. studied the contribution of CAFs to the progress of early-stage CRC (T1CRC) through the co-culture model (Dang et al., 2023). Furthermore, Ramos Zapatero et al. developed a highly multiplexed mass cytometry platform and found that CAFs can regulate PDO plasticity-shifting proliferative colonic stem cells (proCSCs) to slow-cycling revival colonic stem cells (revCSCs) to protect cancer cells from chemotherapy (Ramos Zapatero et al., 2023). The PDO-CAFs model has also been established and applied to pancreatic cancer. Knoblauch M et al. established direct 3D co-cultures of primary PDAC organoids and patient-matched CAFs to investigate the influence of stromal components on chemosensitivity (Knoblauch et al., 2023). Similarly, Grützmeier SE et al. developed a co-culture model using PDTOs and CAFs derived from endoscopic ultrasound-guided fine-needle biopsies (EUS-FNBs) for potential use in drug screening and molecular mechanisms unraveling involved in the chemoresistance-supporting role of the tumor stroma (Grützmeier et al., 2023). In research reported by Zou et al., HCC-PDOs and PBMC are co-cultured with MSC and CAFs to construct HCC organoid-on-a-chip mimicking the original TME (Zou et al., 2023). This TME mimicked novel platform can be applied for high-throughput drug screening and to predict immunotherapy response of HCC patients. All these studies showed that the CRC PDO-CAFs model is suitable for evaluating standard care drugs, making it very useful for achieving personalized cancer drugs.

#### 4.5.2 Co-culture system of PDOs with other immune cells

Co-culture of immune cells with PDO is helpful to further understand the interaction between tumor and immune system, which is of great significance to the research of tumor immunotherapy and can also promote personalized immunotherapy to adapt to reality. In 2018, Dijkstra et al. co-cultured PDO from mismatch repair deficient CRC patients with homologous peripheral blood lymphocytes and successfully enriched tumor-reactive T cells from peripheral blood. This not only realizes the dynamic evaluation of the curative effect of individualized immunotherapy under minimally invasive conditions, but also provides the possibility for adopting T cell therapy with peripheral blood (Dijkstra et al., 2018). Homologously, Cattaneo CM et al. co-cultured peripheral blood lymphocytes with PDO and assessed the generation and function of tumor-reactive T cells in CRC. T cells are evaluated for their capacity to carry out effector functions (IFN-γ secretion and degranulation) after recognition of tumor cells and their capacity to kill tumor organoids (Cattaneo et al., 2020). Besides, Jiang S et al. established a co-culture system with PDOs and macrophages, and Piro G et al. explored a co-culture system with PDOs and T-cells in pancreatic cancer (Piro et al., 2021; Jiang et al., 2023). An *in vitro* organoid/immune cell co-culture model reported by Holokai et al. was cultured to evaluate the specific target mechanisms that deplete MDSCs as a therapeutic strategy for PDAC (Wong et al., 2020). Schnalzger et al. reported a co-culture platform for CRC organoids and CAR-NK cells, which can dynamically and quantitatively monitor CAR-mediated cytotoxicity. The co-culture system showed the targeting effect of CAR cells on tumor-specific antigens and proved that CAR cells can still specifically kill CRC organoids without damaging normal organs under the condition of only a small amount of tumor antigen expression or complex microenvironment (Schnalzger et al., 2019). As described above, the co-culture of CRC organoids and immune cells can not only study the interaction between immune cells and tumors, but also the efficacy of tumor immunotherapy to develop new immunotherapy strategies. However, it has been a problem to be solved urgently: how to simulate the tumor immune microenvironment most realistically and quickly, evaluate the effect of immunotherapy, and screen out the target population that may benefit from itself clinically (Yuki et al., 2020). In addition, the interaction mode between immune cells and tumor cells in TME, the mechanism of directional metastasis of tumor-producing organoids, and the screening and identification of key cell subsets and regulatory molecules that regulate the formation of the metastatic microenvironment also need the support of a reliable co-culture platform *in vitro* (Yang and Yu, 2023).

## 5 Ethical consideration

Human organoids have been widely applied in medicine to extents such as drug development, disease modeling, developmental biology, disease pathology, cell biology, regeneration mechanisms, precision medicine and organ transplantation, which can efficiently simulate the physiological structure, function, development, and similarly maintenance processes of *in situ* tissues, showing great development potential (Tang et al., 2022). With the increasing development and maturity of organoid technology, various organoids, such as the stomach, intestine, liver, retina, brain, and other organoids have been established (Corrò et al., 2020). However, the ethical issues arising from the application are also worth pondering and noting.

### 5.1 Donor’s notification

It is well known that informed consent is necessary to be given to donors when their organs or specimens are donated. When informed consent is signed, the donors, whether have been informed, have the right to accept or reject it, or even have the right to revoke it. In addition, the donors may perceive the application scenarios and procedures for withdrawing informed consent post-sign (Munsie et al., 2017). All the above processes should be strictly implemented and need to be supervised and managed by specialized agencies.

### 5.2 Representativeness of the content of the notice

Organoids are derivatives of the specimens, which are derived from organs, tissues, biopsy specimens, pleural effusion, *etc.*, in clinical assays (Rossi et al., 2018). In general, extensive informed consent was consulted and signed only for source specimens themselves, which is essentially different from the processing products of the source specimens (Boers et al., 2019). However, how to ensure that donors can inform the subsequent possible applications of such specimens in detail, such as successfully cultured organoids for scientific research or clinical utilization of specimen derivatives, remains unclear.

### 5.3 Privacy protection and information disclosure

Today, with the rapid development of various omics such as genomics, proteomics, metabolomics, *etc.*, ethics in the era of big data have been challenged. It is legitimate that, on the premise of patient identity de-associated, human specimens utilized for scientific research after consent informed and confidentiality assurance However, the application of organoids seems to be limited for identity de-association because individual patients who might would be benefit from the study lost the opportunity to obtain experimental treatment. Moreover, the use value and application scenarios of human specimens will be greatly restricted if personal information is disconnected. Therefore, personal information privacy disclosures containing biological information related to biological samples of donors should be appropriately re-discussed.

### 5.4 Regenerative medicine and transplantation of organoids

The wide application of organoids in clinical trials or scientific research lies not only in their ability to highly simulate the physiological structure and function of *in situ* tissues or source organs but also in maintaining the stability and immortalization of genetic information through long-term passaging. Therefore, the application of organoids in regenerative medicine and organ transplantation poses emerging social and ethical challenges (Schneemann et al., 2020). As we all know, although the existing brain organoids have a very low probability of being conscious, how do people know if the brain organoids have developed consciousness (Lavazza and Chinaia, 2023). At present, human brain organoids or other organoids with consciousness or not have been cultivated either intentionally or unintentionally by scientists; the technology for detecting consciousness has not yet been broken through. Therefore, with the continuous development of *in vitro* culture conditions in organoids, people should be prepared for possible risks in brain organoids. When the emergence of stereotyped assembly and fusion technology provides a new probability to generate large-scale organoids, organ transplantation based on organoids will become a reality (Lavazza, 2021). In South Korea and Japan, intestinal organoids have been reported to be used in the clinical treatment of diseases such as ulcerative colitis or refractory Crohn’s disease. However, differences in genetic information between donors and recipients, immune rejection after transplantation, and the degree of benefit and risk to recipients also need to be accurately assessed after transplantation (Sugimoto et al., 2022). At the same time, organ transplantation is also a moral test for donors.

In conclusion, the current ethical norms and regulations need to be revised on a large scale to adapt to the ethical challenges brought by the rapid development of organoid technology and the implementation of clinical trial projects.

## 6 Challenges and prospects

### 6.1 The organoid cultivation system

Firstly, organoid culture technology depends largely on the establishment of the culture system, such as different growth factors and additives added to the medium. Although the organoid culture system of different organs and tissues has been established since the emergence of organoid culture technology, there is still no standard or universal training system or training program.

In the reported culture system, growth factors or additives will make the adapted cells grow preferentially, while the unadapted cells will die during the culture process. Studies have shown that intestinal tumor cells in the organoid culture system maintained the genomic, epigenetic, and transcriptomic characteristics of their in vivo-derived tumor tissues, but the organoid cells had different composition types and different proliferation rates (Wang et al., 2022b).

Secondly, matrigel is also a huge challenge for the stable construction of organoid technology. Matrigel, a basement membrane extract secreted by mouse sarcoma cells, is the most commonly used in organoid culture systems. Due to the unclear composition, heterogeneous characteristics, batch variation, and lack of reproducibility of matrigel, researchers have been committed to exploring alternatives to matrigel. For example, Takahashi et al. successfully used collagen gel instead of matrix gel and improved the medium composition, which was applied to human intestinal organoid culture for drug cytotoxicity screening (Takahashi et al., 2023). Alginate, hydrogel hyaluronic acid, and a mixture of hyaluronic acid and chitosan have also been reported to be good substitutes for matrigel (Sandilya and Singh, 2021; Hillion and Mahe, 2022; Chooi et al., 2023).

Finally, the tumor tissue itself has complex structures and tumor heterogeneity (Gao et al., 2023). In the process of *in vitro* organoid model construction, due to the limitation of the location of tumor tissue, which cannot represent the whole tumor, the limitation of culture conditions, the prolongation of culture time, and the increase of passages, only a part of the tumor cells adapted to the culture conditions, resulting in the surviving organoids losing tumor heterogeneity. In addition, in the process of culture and passage, the proliferation rate of tumor cells is lower than that of normal cells. Therefore, researchers have begun to focus on tumor organoid screening, which may be one of the next research directions (Wallaschek et al., 2019). During long-term *in vitro* culture, new mutations will also accumulate, resulting in increased differences between *in vitro* models and *in vivo* tumors (Yang et al., 2021). Therefore, in the future, more optimized training systems and conditions will be needed to solve these problems.

### 6.2 Culture microenvironment

Tumor stem cells, or epithelial stem cells, were adapted to grow in some specific medium, and matrigel finally formed the organoids. However, these stem cells lacked the *in vivo* tumor microenvironment during the culture process, such as fibroblasts, endothelial cells, immune cells, and other stromal cells and ECM components (Devarasetty et al., 2020). Even different individuals have different tumor microenvironments *in vivo*. However, in the matrigel-coated culture system, the same culture system is used, resulting in a partial lack of heterogeneity. The co-culture of organoids and immune cells is closer to the microenvironment in which cells grow *in vivo*, which is a new direction for the study of tumors and related immunotherapy (Schuth et al., 2022). In addition, the use of organoid microfluidic chip technology seems to be a breakthrough in the study of tumor microenvironment. However, the composition, concentration, and flow rate of multiple cytokines are the bottlenecks to the repeatability of organoid microenvironment reconstruction. In addition, the current organoid models cannot construct well-developed blood vessels, resulting in organoid models that cannot be cultured *in vitro* to a larger size, so the clinical application of organoids is also limited, and the development of engineered vascularization may overcome this problem (Garreta et al., 2021).

## 7 Conclusion

Over all, organoid, as one of the world’s top ten breakthrough technologies, is predicted to be the most promising preclinical disease transformation model in the 21st century. Although organoid technology currently faces considerable difficulties to be accomplished, its accurate simulation of human tumor characteristics still shows great potential for preclinical application. We all believed that, with the further precision of ethical recognition, the continuous maturity of organoid technology, and the solution of the microenvironment culture scheme, future efforts will undoubtedly bring this new technology closer to clinical practice.

## Figures and Tables

**Table:** 

**PDOs**	patient-derived organoids
**GI**	gastrointestinal
**GC**	gastric cancer
**CRC**	colorectal cancer
**PDAC**	pancreatic ductal adenocarcinoma
**MAD4**	max dimerization protein 4
**TP53**	tumor protein 53
**DCRC**	deleted in colorectal cancer
**APC**	adenomatous polyposis coli
**KRAS**	kirsten rats arcomaviral oncogene homolog
**HER2**	human epidermal growth factorreceptor 2
**C-MYC**	myelocytomatosis viral oncogene homolog
**miRNA**	microRNA
**CRISPR/Cas9**	clustered regularly interspaced short palindromic repeats and associated protein 9
**SMAD4**	mothers against decapentaplegic homolog 4
**3D**	three-dimensional
**PDX**	patient-derived xenograft
**PTC**	patient-derived tumor-like cell cluster
**PD-L1**	programmed cell death ligand 1
**ECM**	extracellular matrix
**PSCs**	pluripotent stem cells
**ESCs**	embryonic stem cells
**hiPSC**	human induced pluripotent Stem cells
**ASCs**	adult stem cells
**Lgr5+**	leucine-rich repeat-containing G-protein-coupled receptor 5-positive
**EGF**	epidermal growth factor
**BMP**	bronchial mucous proteinase
**sgRNA**	small guide RNA
**PAM**	proto spacer adjacency motif
**DACH1**	dachshund homologue 1
**LARGE2**	like glycosyltransferase 2
**KDM1A**	lysine-specific histone demethylase 1A
**FGFR1**	fibroblast growth factor receptor 1
**OXTR**	oxytocin receptor
**ESCs**	embryonic stem cells
**CDKN2A**	cyclin dependent kinase inhibitor 2A
**ARID1A**	AT-rich interactive domain-containing protein 1A
**TDD**	targeted drug discovery
**PDD**	phenotype drug discovery
**SRCC**	sig-ring cell carcinoma
**MSC**	mesenchymal stromal cell
**PBMC**	peripheral blood mononuclear cell
**CAF**	cancer-associated fibroblasts
**TME**	tumor microenvironment
**PGE2**	prostaglandin E2
**PGD**	phosphogluconate dehydrogenase
**C8orf76**	chromosome 8 open reading frame 76
**lncRNA**	long intergenic non-coding RNA
**MAPK**	mitogen-activated protein kinase
**PKM2**	pyruvate kinase M2 isoform
**PRMT6**	protein arginine N-methyltransferase 6
**PKM2**	pyruvate kinase M2 isoform
**ATG5**	autophagy Protein 5
**WTAP**	wilms tumor 1 associated protein
**HCC**	hepatocellular carcinoma
**BRAF**	v-raf murine sarcoma viral oncogene homolog B
**ICC**	intrahepatic cholangiocarcinoma
**VEGF**	vascular endothelial growth factor
**EpCAM**	epithelial cell adhesion molecule
**MCLl**	myeloid cell leukemia-1
**BDNF**	brain derived neurotrophic factor
**MYOF**	myofibroblast
**PRMT6**	protein arginine N-methyltransferase 6
**YAP1**	yes-associated protein 1
**proCSCs**	proliferative colonic stem cells
**revCSCs**	revival colonic stem cells
**IFN-γ**	interferon γ
**MDSCs**	myeloid-derived suppressor cells
