# Supplementary material for: Human patient derived organoids: an emerging precision medicine model for gastrointestinal cancer research
Source: Front Cell Dev Biol. 2024 Apr 4;12:1384450. doi: 10.3389/fcell.2024.1384450 (PMC11024315; doi:10.3389/fcell.2024.1384450)
Supplement: Supplementary file 1 [file Table1.DOCX]

**Table 1 Comparison of tumor models**

| Model | Cell line | PDX | PDO | PTC |
| --- | --- | --- | --- | --- |
| Cost | **+** | **+++** | **++** | **++** |
| Time consumption | **+** | **+++** | **++** | **++** |
| Success rate | **+++** | **+** | **++** | **++** |
| Genetic modification | **+++** | **+** | **++** | **-** |
| Genetic background retention | **-** | **+++** | **+++** | **+++** |
| Tumor heterogeneity | **-** | **++** | **+** | **+** |
| Drug efficacy prediction | **-** | **++** | **++** | **++** |
| High-throughput drug testing | **+++** | **-** | **++** | **++** |
| Personalized therapy | **-** | **++** | **+++** | **+++** |
| TME interaction research | **-** | **+++** | **++** | **++** |
| lmmunotherapy evaluation | **-** | **+++** | **++** | **++** |
| Biobanking | **-** | **-** | **+++** | **-** |

PDX: patient-derived xenograft, PDO: patient-derived organoid, TME: tumor microenvironment, +: low correlation, ++: medium correlation, +++: high correlation, -: not suitable
