# Supplementary material for: Human patient derived organoids: an emerging precision medicine model for gastrointestinal cancer research
Source: Front Cell Dev Biol. 2024 Apr 4;12:1384450. doi: 10.3389/fcell.2024.1384450 (PMC11024315; doi:10.3389/fcell.2024.1384450)
Supplement: Supplementary file 2 [file Table2.DOCX]

|  | DATE | Target | Type of gene editing | Organoid | Xenotransplantation of organoids | | Function | Ref |
| --- | --- | --- | --- | --- | --- | --- | --- | --- |
| 1 | 2020 | DACH1 | knock out | CRC organoids | / | the importance of DACH1 for CRC organoid formation and stemness | | 74 |
| 2 | 2020 | LARGE2 | knock out | human colon tumor organoid | / | silenced LARGE2 compromised O-glycosylation of α-DG in CRC | | 75 |
| 3 | 2022 | KIT | knock out | CRC Organoid | NSG | caused a partial mesenchymal-to-epithelial phenotype switch and a strong reduction of intra-tumor stromal content | | 76 |
| 4 | 2023 | KDM1A-KO | Epigenetic library for negative select screen | GC PDOs | / | assess the contribution of epigenetic regulators to gastric cancer | | 77 |
| 5 | 2016 | KRAS^G12D^ | knock out | patient-derived CRC organoid | / | evaluated RAS pathway inhibitors and drug combinations that are currently in clinical trial for RAS mutant cancers in vitro | | 78 |
| 6 | 2021 | KRAS^G12D^ | knock out | CRC organoids | / | specific effect of KRASG12D acquisition in drug-tolerant organoids. | | 79 |
| 7 | 2022 | SMAD4^R361H^ | knock out | CRC organoids | / | drug screening and reverses MEK-inhibitor resistance | | 80 |
| 8 | 2019 | FGFR1 or OXTR | knockout | colon cancer organoids | / | help overcome oxaliplatin resistance | | 81 |
| 9 | 2018 | GATA6，KCTS | knock out | PDAC organoids | NOD.Cg-*Prkdc*^scid^Il2rg*^tm1Sug^*/Jic(NOG) | demonstrated the stepwise tumorigenesis of PDAC with progressive acquisition of niche independency. | | 82 |
| 10 | 2022 | ARID1A, BRCA2. | knock out | human PDAC organoid | / | drug screen for identifying candidates | | 83 |
| 11 | 2017 | LGR5‐EGFP and KI67‐TagRFP2 | knock in | CRC organoids | NOD/SCID female mice | integrate reporter cassettes at desired marker genes | | 84 |

PDOs: patient-derived organoids, CRC: colorectal cancer, GC: gastric cancer, PDAC: pancreatic ductal adenocarcinoma
