# Supplementary material for: Human patient derived organoids: an emerging precision medicine model for gastrointestinal cancer research
Source: Front Cell Dev Biol. 2024 Apr 4;12:1384450. doi: 10.3389/fcell.2024.1384450 (PMC11024315; doi:10.3389/fcell.2024.1384450)
Supplement: Supplementary file 3 [file Table3.DOCX]

| Register ID |  | Status | Brief title | Estimated enrollment | Population | Intervention | Outcome | Study design | Nation | Registration date |
| --- | --- | --- | --- | --- | --- | --- | --- | --- | --- | --- |
| NCT05401318 |  | Recruiting | Tailoring Treatment in Colorectal Cancer (Target CRC) | 40 | Patients with suspected colorectal cancer referred into the program for standardized cancer diagnostic pathways at Akershus University Hospital. | Tumor tissue sampling for organoid development | identification of chemotherapy or chemotherapy combinations with or without targeted therapies that induce immunotherapy efficacy in CRC | Observational | Norway | 2022/3/28 |
| NCT04996355 |  | Recruiting | Organoids-on-a-chip for Colorectal Cancer and in Vitro Screening of Chemotherapeutic Drugs | 52 | patients with advanced colorectal cancer | Other: comprehensive treatment after MDT discussion | the accuracy, specificity and sensitivity of organoids-on-chip for drug sreening | Observational | China | 2021/8/2 |
| NCT06196554 |  | Recruiting | Gastric Cancer Organoids in the Screening of Neoadjuvant Drugs | 40 | patients with locally advanced gastric cancer receiving neoadjuvant chemotherapy. | Drug: Oxaliplatin | the proportion of inconsistencies between organoid drug screening results and actual clinical observation drug sensitivity | Observational | China | 2023/12/1 |
| NCT06100016 |  | Recruiting | A Clinical Study Aims to Assess the Consistency of Clinical Efficacy in Colorectal Cancer Treatment and Drug Susceptibility Outcomes Using a Novel Drug Susceptibility Testing Method | 105 | patients with colorectal cancer in the First Affiliated Hospital of China Medical University | Not provided | evaluate the consistency of clinical efficacy in colorectal cancer treatment and drug susceptibility outcomes. | Observational | China | 2023/10/20 |
| NCT06100003 |  | Recruiting | A Clinical Study Aims to Assess the Consistency of Clinical Efficacy in Gastric Cancer Treatment and Drug Susceptibility Outcomes Using a Novel Drug Susceptibility Testing Method | 104 | patients with gastric cancer in the First Affiliated Hospital of China Medical University | Not provided | evaluate the consistency of clinical efficacy in gastric cancer treatment and drug susceptibility outcomes. | Observational | China | 2023/10/25 |
| NCT03577808 |  | NOT YET RECRUITING | Organoids in Predicting Chemoradiation Sensitivity on Rectal Cancer | 80 | Organoids in Predicting Chemoradiation Sensitivity on Rectal Cancer | Other: Biopsy | predict the clinical outcome in locally advanced rectal cancer patients underwent neoadjuvant chemoradiation. | Observational | China | 2018/7/5 |
| NCT04906733 |  | NOT YET RECRUITING | Cetuximab Sensitivity Correlation Between Patient-Derived Organoids and Clinical Response in Colon Cancer Patients. | 80 | Patients with recurrent and metastatic colorectal cancer | Procedure: Tumor biopsy | to assess the consistency of organoid chemotherapy sensitivity and clinical chemotherapy efficacy | Observational | China | 2021/5/28 |
| NCT04497727 |  | NOT RECRUITING | Gut Organoid Study | 41 | Hypertensive and normotensive patients who routinely undergo scheduled elective colonoscopy | Other: Gene Expression | compare basic properties of gut epithelia of hypertensive and normotensive reference subjects. | Observational | United States | 2020/8/4 |
| NCT05832398 |  | Recruiting | Precision Chemotherapy Based on Organoid Drug Sensitivity for Colorectal Cancer | 186 | Colorectal Cancer | Drug: FOLFOX , FOLFIRI or FOLFOXIRI regimens | to investigate whether chemotherapy guided by PDOs drug test can improve the outcomes of stage IV CRC. | Interventional | China | 2023/4/27 |
| NCT05294107 |  | Recruiting | Intestinal Organoids (BIOÏDES) | 90 | Digestive System Diseases | Procedure: additional biopsies | generate a biocollection of 3D intestinal models from digestive biopsies with associated health data and to characterize them before using them for the screening of potential therapeutic molecules. | Interventional | France | 2022/3/24 |
| NCT05038358 |  | NOT YET RECRUITING | Tumor Immune Microenvironment Involvement in Colorectal Cancer Chemoresistance Mechanisms (CRC-ORGA 2) | 60 | colorectal adenocarcinoma, naive from neo-adjuvant chemotherapy, indication of surgical resection | Not provided | develop a model of tumoroids derived from patients with a colorectal tumors prior to any systemic anti cancer treatment. | Observational | France | 2021/9/9 |
| NCT02874365 |  | COMPLETED | Intestinal Stem Cells Characterization (BIODIGE) | 110 | Inflammatory Bowel Diseases | Inflammatory Bowel Diseases | investigate the morphological characteristics of organoids, the expression of genes and proteins of the Wnt/APC/beta-catenin pathway within both ISC. | Interventional | France | 2016/8/22 |
| NCT04371198 |  | COMPLETED | Patient-Derived Organoids for Rectal Cancer | 20 | Rectum Cancer | Other: Biopsy | to determine the feasibility of establishing patient-derived organoids from pre-treatment rectal adenocarcinoma biopsies. | Interventional | United States | 2020/5/1 |
| NCT03256266 |  | Recruiting | Effect of Antigens or Therapeutic Agents on in Vitro Human Intestinal Organoids | 375 | Patients with patients with Food intolerances, Food allergy, intestinal disorders, glutensensitivity, healthy controls who underwent a gastroduodenoscopy or coloscopy for therapeutical intervention or screening | Not provided | the study evaluates the effect of nutrient antigens or therapeutic agents on human small intestinal organoids. | Observational | Germany | 2017/8/22 |
| NCT05351398 |  | NOT YET RECRUITING | The Clinical Efficacy of Drug Sensitive Neoadjuvant Chemotherapy Based on Organoid Versus Traditional Neoadjuvant Chemotherapy in Advanced Gastric Cancer | 54 | Patients with advanced gastric cancer whose tumor is located in the stomach and need neoadjuvant therapy before radical surgery. | Drug: PDO group Drug: PDO group | access the safety and clinical value of the personalized neoadjuvant therapy based on patient-derived organoid drug sensitivity assay in advanced gastric cancer. | Observational | China | 2022/4/28 |
| NCT05352165 |  | NOT YET RECRUITING | The Clinical Efficacy of Drug Sensitive Neoadjuvant Chemotherapy Based on Organoid Versus Traditional Neoadjuvant Chemotherapy in Advanced Rectal Cancer | 192 | Neoadjuvant Therapy | Drug: standard long-term therapy | the organoid sample bank of more than 100 patients with locally advanced rectal cancer was completed | Interventional | Not provided | 2022/4/28 |
| NCT05183425 |  | Recruiting | Patient-derived Organoids Predicts the Clinical Efficiency of Colorectal Liver Metastasis | 60 | All patients with histologically proven colorectal cancer with liver metastasis. | Not provided | investigate the consistency of drug sensitivity for the matched primary and metastatic tumor in patients with liver metastasis. | Observational | China | 2022/1/10 |
| NCT05203549 |  | UNKNOWN STATUS | Consistency Between Treatment Responses in PDO Models and Clinical Outcomes in Gastric Cancer | 250 | Two hundred and fifty patients with gastric cancer who need to receive neoadjuvant therapy, conversion therapy or palliative chemotherapy are included in this study | Procedure: Tumor biopsy | to evaluate the consistency between treatment response in the PDO model and patient clinical outcomes | Observational | China | 2022/1/24 |
| NCT05425901 |  | Recruiting | Preclinical Evaluation of Multimodal Therapeutic Strategies in Intestinal Irradiation and Inflammatory Bowel Disease From Organoids (INTRUST) | 80 | Radiation Enteritis Radiation Enteritis Inflammatory Bowel Diseases | Other: biopsy | to setup a screening tool by irradiating the organoids (step one) and then evaluate in vitro the regenerative activity of treatments dedicated to improve inflammatory bowel diseases and acute radiation enteritis | Interventional | France | 2022/6/21 |
| NCT05304741 |  | Recruiting | The Culture of Advanced/​Recurrent/​Metastatic Colorectal Cancer Organoids and Drug Screening | 30 | Patients suffered from advanced/recurrent/metastatic colorectal cancer which was diagnosed as adenocarcinoma and unresectable. | Not provided | organoids from patients with advanced/relapsed/metastatic colorectal cancer are established, and drug screening assays for organoids will be performed with chemotherapy and targeted agents and compared with clinical practice | Observational | China | 2022/3/31 |
| NCT05652348 |  | Recruiting | Response Prediction of Hyperthermic Intraperitoneal Chemotherapy in Gastro- Intestinal Cancer (Hi-STEP1) | 48 | Eligible patients are recruited consecutively during of the initial surgical treatment planning | Other: Establishment of organoid cultures and in vitro sensitivity testing | organoid cultures from biopsies were established, various chemotherapeutic agents were tested on these tumor organoids, and genetic changes in tumor organoids were analyzed | Observational | Germany | 2022/12/15 |
| NCT06057298 |  | Recruiting | Patient-tailored Hyperthermic Intraperitoneal Chemotherapy (HIPEC) for Colorectal Peritoneal Metastases | 24 | Peritoneal Metastases From Colorectal Cancer | Procedure: Patient-tailored HIPEC | to demonstrate that cytoreductive surgery and patient-tailored HIPEC will increase efficacy in controlling peritoneal disease | Interventional | Italy | 2023/9/28 |
| NCT05883683 |  | NOT YET RECRUITING | Molecular Study and Precision Medicine for Colorectal Cancer (MSPM) | 100 | patients with advanced or recurrent colorectal cancer | Other: Molecular Profiling & drug testing in tumor organoids and PDXs | to identify clinical actionable targets and predict in vivo response of the tumor to targeted drugs by using PDOs/ PDXs | Observational | China | 2023/6/1 |
| NCT05725200 |  | Recruiting | Study to Investigate Outcome of Individualized Treatment in Patients With Metastatic Colorectal Cancer (EVIDENT) | 40 | Metastatic Colorectal Cancer | Drug: Alectinib、Cetuximab、Crizotinib | to investigate the effect and side effects of personalized cancer treatment in patients with metastatic colorectal cancer (bowel cancer) | Interventional/Phase 2 | Norway | 2023/2/13 |
| NCT03283527 |  | Recruiting | Organoid Based Response Prediction in Esophageal Cancer (RARESTEM/Org) | 100 | patients with curatively treatable and resectable esophageal cancer | Not provided | the steepness of the dose response survival curve in the organoids in relation to the pathologic response after resection in the clinical situation. | Observational | Netherlands | 2017/9/12 |
| NCT05644743 |  | NOT YET RECRUITING | Clinical Transformation of Organoid Model to Predict the Efficacy of GC in the Treatment of Intrahepatic Cholangiocarcinoma | 40 | patients were histologically or cytologically diagnosed with locally advanced inoperable radical resectable or metastatic intrahepatic cholangiocarcinoma. | gemcitabine + cisplatin | build an organoid-based drug resistance prediction model. | Observational | Not provided | 2022/11/30 |
| NCT04931394 |  | Recruiting | Organoid-Guided Adjuvant Chemotherapy for Pancreatic Cancer | 200 | Pancreatic Cance | Other: Adjuvant chemotherapy guided by organoid drug sensitivity test | the consistency between the drug sensitivity test results and the treatment response of patients will be analyzed | Interventional/Phase 3 | China | 2021/6/18 |
| NCT04931381 |  | Recruiting | Organoid-Guided Chemotherapy for Advanced Pancreatic Cancer | 100 | Advanced Pancreatic Cancer | Other: Chemotherapy guided by organoid drug sensitivity test | explore the concordance between drug sensitivity test results and patients' treatment response. | Interventional/Phase 3 | China | 2021/6/18 |
| NCT05196334 |  | NOT RECRUITING | Pharmacotyping of Pancreatic Patient-derived Organoids | 88 | Patients with histopathological confirmation of pancreatic ductal adenocarcimona, ineligible for surgery, planned to start standard first line treatment | Other: No intervention | to use organoids cultured from diagnostic endoscopic ultrasound (EUS)-guided fine needle biopsy (FNB) samples from patients with PDAC for pharmacotyping. | Observational | Denmark | 2022/1/19 |
| NCT05842187 |  | Recruiting | In Vitro Organoid Drug Sensitivity-Guided Treatment for Metastatic Pancreatic and Gastric Cancer (ODYSSEY) | 20 | Pancreatic Cancer Gastric Cancer | Procedure: Biopsy of tumor tissue for organoid culture | to evaluate the consistency between in vitro tumor organoid drug sensitivity and the therapeutic efficacy of in vivo drug treatment. | Interventional | China | 2023/5/3 |
| NCT05351983 |  | Recruiting | Patient-derived Organoids Drug Screen in Pancreatic Cancer | 50 | Pancreas Cancer | Procedure: Surgical biopsy of tumoral tissue for organoid generation | help to speed up the implementation of organoid generation in the clinical routine for the choice of the best treatment of patients affected by pancreatic cancer. | Interventional | Switzerland | 2022/4/28 |
| NCT05634694 |  | Recruiting | Study on Consistency Evaluation for Drug Sensitivity of Patient-Derived Organoid Model From Cholangiocarcinoma Patients | 40 | Histologically confirmed cholangiocarcinoma patients who underwent radical resection and received post-operation adjuvant chemotherapy. | Other: No interventions | evaluate the consistency and accuracy of patient-derived organoid model of cholangiocarcinoma to predict the clinical chemotherapeutic efficacy, as well as the possibility of guiding the adjuvant chemotherapy. | Observational | China | 2022/12/2 |
| NCT04777604 |  | NOT YET RECRUITING | Development of a Prediction Platform for Neoadjuvant Treatment and Prognosis in Pancreatic Cancer Using Organoid | 300 | patient who have been diagnosed with pancreatic cancer via EUS-FNA and EUS-FNB at Samsung Medical Center. | Other: Organoid | to investigate the relationship between unique genomic mutations and responsiveness to anti-cancer drugs in patients with pancreatic cancer | Observational | Korea | 2021/3/2 |
| NCT03500068 |  | UNKNOWN STATUS | Establishing Organoids From Metastatic Pancreatic Cancer Patients, the OPT-I Study. (OPT-1) | 30 | Carcinoma, Pancreatic Ductal | Carcinoma, Pancreatic Ductal | to develop a model system and infrastructure to individualize the treatment of patients with advanced pancreatic adenocarcinoma | Interventional | Netherlands | 2018/4/17 |
| NCT04736043 |  | Recruiting | Development of a Prediction Platform for Adjuvant Treatment and Prognosis in Resected Pancreatic Cancer Using Organoid | 300 | All adult patients (> 18 years) with (a suspicion of) advanced pancreatic adenocarcinoma | Other: Organoid | to investigate the relationship between unique genomic mutations and responsiveness to anti-cancer drugs in patients with pancreatic cancer | Observational | Korea | 2021/2/3 |
| NCT03544255 |  | UNKNOWN STATUS | Drug Screening of Pancreatic Cancer Organoids Developed From EUS-FNA Guided Biopsy Tissues | 50 | Patients suspected to have pancreatic cancer who are going to receive EUS-FNA operations for diagnostic purposes. | Procedure: Biopsy | establish organoid models from pancreatic cancer biopsies achieved via EUS-FNA | Observational | China | 2018/6/1 |
| NCT05932836 |  | Recruiting | An Organoid-on-chips Technique Based on Biopsy Samples and Its Efficacy in Predicting the Response to HAI in HCC | 165 | Patiens who need to undergo biopsy for malignant tumors such as breast cancer, lung cancer, liver cancer, bile duct cancer or pancreatic cancer that have been clinically and/or pathologically diagnosed. | Other: No interventions | to evaluate the predicting efficacy of the established organoid-on-chips system in HCC patients who undergo hepatic artery infusion (HAI) with mFOLFOX6. | Observational | China | 2023/7/6 |
| NCT03990675 |  | UNKNOWN STATUS | Evaluation and Comparison of the Growth Rate of Pancreatic Cancer Patient-derived Organoids | 50 | Patients with indication for EUS-guided FNA or FNB of a suspected pancreatic malignancy Exclusion Criteria | Procedure: FNA, FNB | evaluate and compare the growth rate of pancreatic cancer patient-derived organoids generated from matched fine needle Aspirations (FNA) and fine needle biopsies (FNB). | Interventional | Germany | 2019/6/19 |
| NCT05571956 |  | Recruiting | Establishment of Pancreas Cancer and Cancer-associated Fibroblast Using EUS-guided Biopsy Samples | 50 | Pancreas Adenocarcinoma | Other: Pancreatic ductal adenocarcinoma organoids and cancer-associated fibroblasts | simultaneously establish the patient-derived PDA organoids as well as CAFs using EUS-FNB samples. | Interventional | Korea | 2022/10/7 |
| NCT02436564 |  | UNKNOWN STATUS | In Vitro Models of Liver and Pancreatic Cancer | 75 | Patients who are undergoing surgical resenctions of liver, biliary or pancreas cancers that are able to give informed consent | Genetic: Organoid in vitro culture | develop an in vitro model of cancer for laboratory study using liver, biliary and pancreatic cancer tissue | Observational | United Kingdom | 2015/5/7 |

PDOs: patient-derived organoids, GI: gastrointestinal, CRC: colorectal cancer, HIPEC: Hyperthermic Intraperitoneal Chemotherapy, PDAC: pancreatic ductal adenocarcinoma
